# Computational fluid dynamics benchmark dataset of airflow in tracheas

**DOI:** 10.1016/j.dib.2016.11.091

**Published:** 2016-11-28

**Authors:** A.J. Bates, A. Comerford, R. Cetto, D.J. Doorly, R.C. Schroter, N.S. Tolley

**Affiliations:** a Department of Aeronautics, Imperial College, London, United Kingdom; b Department of Otolaryngology, St. Mary׳s Hospital, Imperial College Healthcare Trust, United Kingdom; c Department of Bioengineering, Imperial College, London, United Kingdom

**Keywords:** Tracheas, CFD, Airflow, Goiters, LES, DNS

## Abstract

Computational Fluid Dynamics (CFD) is fast becoming a useful tool to aid clinicians in pre-surgical planning through the ability to provide information that could otherwise be extremely difficult if not impossible to obtain. However, in order to provide clinically relevant metrics, the accuracy of the computational method must be sufficiently high. There are many alternative methods employed in the process of performing CFD simulations within the airways, including different segmentation and meshing strategies, as well as alternative approaches to solving the Navier–Stokes equations. However, as *in vivo* validation of the simulated flow patterns within the airways is not possible, little exists in the way of validation of the various simulation techniques. The data presented here consists of very highly resolved flow data. The degree of resolution is compared to the highest necessary resolutions of the Kolmogorov length and time scales. Therefore this data is ideally suited to act as a benchmark case to which cheaper computational methods may be compared. A dataset and solution setup for one such more efficient method, large eddy simulation (LES), is also presented.

**Specifications Table**

**Table:** 

Subject area	Physics
More specific subject area	Fluid mechanics & Biomechanics
Type of data	Table, Figures, CSV file & STL file
How data was acquired	Computed tomography (CT) segmentation and Computational Fluid Dynamics (CFD) simulation
Data format	STL file, CSV file & Analyzed data
Experimental factors	Airway surface data from segmented CT data
Experimental features	Flow data presented at near direct numerical simulation resolution.
Data source location	N/A
Data accessibility	Data available with article

**Value of Data**

• The tracheal geometry can act as a benchmark geometry for assessment of computational methods for airflow in the tracheal region.
• The extracted data (locations of extraction given) and associated refinement statistics have been provided from the DNS simulation. Researchers can compare their methods precisely with the provided case at the same locations.
• A detailed description of methods for calculating turbulent refinement statistics are outlined for future investigations.
• UA provides a simple measure for the degree of non-uniformity in the flow and is particularly useful for flow in constricted and curved tubular geometries. Benchmark values of this metric are provided in this paper.

## 1 Data

The dataset includes: the tracheal geometry in STL format (Supplementary 1) and a CSV file of points that represent the centerline (Supplementary 2); extracted fluid mechanical metrics and turbulent statistics (including locations of extraction) from a simulation approaching the level of DNS. Additionally, all methods to calculate fluid mechanical metrics are outlined, including turbulent statistics (Section 4.2) and UA (Section 4.3). UA data has been provided for a number of idealised flow scenarios.

## 2 Experimental design, materials and methods

### 2.1 Reference and LES validation data

Two different tracheal airflow datasets are presented: a reference solution and a large eddy simulation. The reference solution is near DNS and is computationally expensive as it tries to resolve all scales, while LES is cheaper as it introduces a model that represents the effect of the unresolved scales on the resolved scales. As the reference solution is near DNS it can be used as benchmark data to assess the validity of a turbulence model, such as LES, and also to assess the degree of turbulence within the flow.

**Fig. 1:**
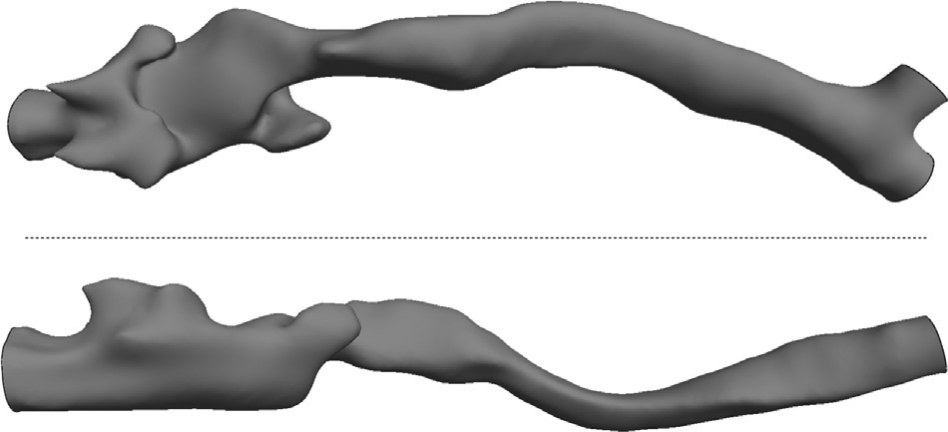
Validation geometry for use as a benchmark case for airflow in pathological tracheas. The geometry in STL format is given in Supplementary 1. Top coronal view and bottom sagittal view.

Analysis of convergence data was performed on one geometry (case B in Bates et al. [1] and shown in Fig. 1). The data was extracted from this geometry as it demonstrates large curvature and constriction. Hence represents a challenging benchmark from a flow computation point of view. A highly resolved simulation, with 9.2 million elements and a time step of 0.01 ms was performed as a reference case and was found to be approaching the resolution required for direct numerical simulation, as shown in Table 1. The Kolmogorov length and time scales were calculated as described in Section 4.2 and the dissipation values did not change significantly with refinement level, suggesting these values had converged. Therefore, the estimates of Kolmogorov scales were deemed sufficiently accurate. The cube root of cell volume, $3\sqrt{V}$ and the time step ∆t were used as characteristic length and time scales respectively. The ratio $3\sqrt{V}/\eta$ represents the mesh refinement level divided by the smallest length scale in the flow. The parameter $∆t/\tau_{\eta }$ is the ratio between the time step and the smallest time scale it is necessary to resolve, neglecting convection, while UΔt/η shows how far such a feature can be convected during one time step as a ratio to the smallest length scale in the flow.

The highly resolved data was used as a reference case to which reduced resolution data could be compared. Simulation meshes were generated with numbers of elements ranging from 945,000 to 9.2 million and quasi-steady simulations were performed on each mesh with time steps of 1, 0.1 and 0.01 ms. These coarser simulations were run both without further turbulence modelling and with the LES model.

**Fig. 2:**
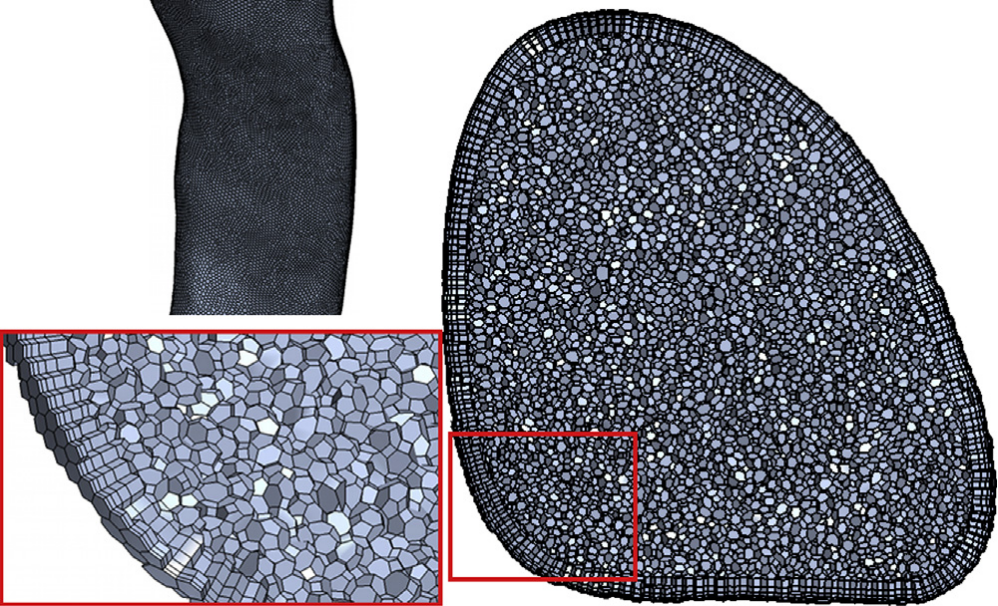
Details of the LES mesh structure. Clockwise from top left: the surface of the trachea; all mesh elements that fall on a cross-sectional plane; a detail highlighting the prism layer mesh at the geometry wall.

**Fig. 3:**
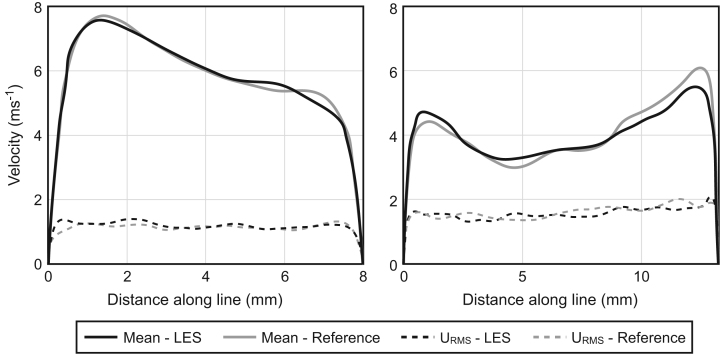
Mean and fluctuating velocity data extracted on lines across the trachea normal to the flow path for the LES and Reference meshes. The Cartesian coordinates of the beginning and end points of the lines are (148.63, 131.14, −162.03) to (156.82, 134.21, −158.17) and (142.82, 114.12, −139.45) to (156.16, 114.54, −140.37) for the left and right figures, respectively.

The parameters found to offer the best compromise between matching the benchmark data and computational expense were a mesh of 2.2 million elements, with five prism layers starting at 0.15 mm high and increasing with a geometric progression of 1.3, a time step of 0.1 ms and with LES turbulence modelling. This mesh is shown in Fig. 2. Several criteria were used to judge the data: convergence, such as mean and fluctuating (see definitions in Section 4.2); velocity along lines normal and tangential to the flow path, as shown in Fig. 3; overall mean pressure drop from the glottis to one diameter above the apex of the bifurcation at the carina; as well as the amplitude and frequency of pressure oscillations. Table 2 shows these values to be close to identical for the reference and LES simulations. Each LES simulation took between 2800 and 4000 core hours to model the required period of inhalation. This time makes running large data sets at different flow rates prohibitively expensive.

**Fig. 4:**
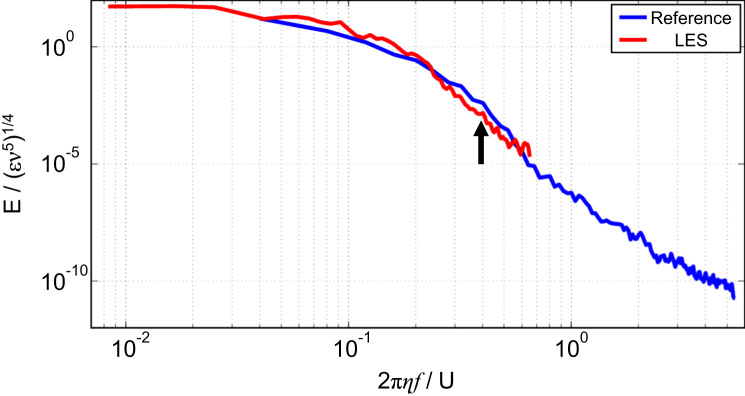
Fluctuating velocity spectra data at the same point in the Reference and LES simulation. The energy is scaled by the dissipation, *ε* and the kinematic viscosity, *ν*. The frequency, *f* is scaled by the mean velocity at the point, *U* and the Kolmogorov length scale, *η*. The arrow points to the value of Δ*w*, defined in Eq. (1).

Fig. 4 shows spectra of fluctuating velocity data at the same point in both the Reference and LES simulations. The spectra are calculated using the method described in Varghese et al. [6], but using just two Hann windows, due to the limited sample size. The spectra are then normalised as described by Saddoughi and Veeravalli [4] for comparison with wave number spectra. The data for the two resolutions reveals that the LES turbulence modelling extends to the scales where the fluctuating energy has fallen by five orders of magnitude, so is within the inertial range. The local grid filter used for the LES simulations was defined as

(1) $$\Delta_{w}=C_{w}V^{\frac{1}{3}}$$

where $C_{w}=0.544$ and V is the cell volume.

### 2.2 Turbulent statistics

This section provides data on important turbulent statistics provided with the dataset.

The Kolmogorov length and time scales are both calculated from the dissipation, ε which is defined by Delafosse et al. [2] as

(2) $$\varepsilon =\nu \left\{2\left(\bar{{\left(\frac{\partial u_{1}^{\prime }}{\partial x_{1}}\right)}^{2}}+\bar{{\left(\frac{\partial u_{2}^{\prime }}{\partial x_{2}}\right)}^{2}}+\bar{{\left(\frac{\partial u_{3}^{\prime }}{\partial x_{3}}\right)}^{2}}\right)+\bar{{\left(\frac{\partial u_{1}^{\prime }}{\partial x_{2}}\right)}^{2}}+\bar{{\left(\frac{\partial u_{2}^{\prime }}{\partial x_{1}}\right)}^{2}}+\bar{{\left(\frac{\partial u_{1}^{\prime }}{\partial x_{3}}\right)}^{2}}+\bar{{\left(\frac{\partial u_{3}^{\prime }}{\partial x_{1}}\right)}^{2}}+\bar{{\left(\frac{\partial u_{2}^{\prime }}{\partial x_{3}}\right)}^{2}}+\bar{{\left(\frac{\partial u_{3}^{\prime }}{\partial x_{2}}\right)}^{2}}+2\left(\bar{\frac{\partial u_{1}^{\prime }}{\partial x_{2}}\frac{\partial u_{2}^{\prime }}{\partial x_{1}}}+\bar{\frac{\partial u_{1}^{\prime }}{\partial x_{3}}\frac{\partial u_{3}^{\prime }}{\partial x_{1}}}+\bar{\frac{\partial u_{2}^{\prime }}{\partial x_{3}}\frac{\partial u_{3}^{\prime }}{\partial x_{2}}}\right)\right\}$$

where $u_{1}^{\prime }$, $u_{2}^{\prime }$ and $u_{3}^{\prime }$ represent the components of fluctuating velocity and νthe kinematic viscosity. The Kolmogorov length scale, η is then calculated by

(3) $$\varepsilon =\nu \eta ={\left(\frac{\nu^{3}}{\varepsilon }\right)}^{\frac{1}{4}}$$

and the Kolmogorov time scale, $\tau_{\eta }$ by

(4) $$\tau_{\eta }={\left(\frac{\nu }{\varepsilon }\right)}^{\frac{1}{2}}.$$

To interpret turbulent quantities, averaging operations are utilised. For a specific flow variable (f) the time average is defined by,

(5) $$\langle f\rangle =\frac{1}{T}\int_{t}^{t+T}f\left(x,y,z,t\right)\mathrm{dt},$$

where T is the time period over which the variable is sampled. Numerically this procedure is defined by

(6) $$\langle f(x,y,z,t)\rangle =\frac{1}{N}\sum_{i=0}^{N}f_{i}\left(x,y,z,t\right),$$

where N indicates the number of samples taken over the period T. Random turbulent fluctuations (f′) are represented by the instantaneous deviation from this mean value defined by

(7) $$f^{\prime }\left(x,y,z,t\right)=f\left(x,y,z,t\right)-\langle f\left(x,y,z,t\right)\rangle .$$

Using Eq. (7) the variance of these fluctuations is defined by

(8) $$\mathrm{Var}\left(f^{\prime }\right)=\langle {f\prime \left(x,y,z,t\right)}^{2}\rangle =\langle {f\left(x,y,z,t\right)}^{2}\rangle -{\left(f\left(x,y,z,t\right)\right)}^{2}$$

Numerically Eq. (8) can be expressed by

(9) $$\mathrm{Var}\left(f^{\prime }\right)=\frac{1}{N-1}\left[\sum_{i=1}^{N}f_{i}^{2}-\frac{1}{N}{\left(\sum_{i=1}^{N}f_{i}\right)}^{2}\right]$$

which represents the square of the root mean square value ($f_{\mathrm{rms}}^{\prime }$), which is commonly quoted in literature.

The turbulent kinetic energy is related to the velocity fluctuations by

(10) $$\mathrm{TKE}=\frac{1}{2}\left({\langle u_{x}^{\prime }\rangle }^{2}+{\langle u_{y}^{\prime }\rangle }^{2}+{\langle u_{z}^{\prime }\rangle }^{2}\right)$$

As an example of typical averaging windows, in Bates et al. [1] all computations were simulated for 0.25 s of steady inhalation. Of this period, the first 0.1 s was ignored, to allow for transients caused by the impulsive start to die away. This period was determined by analysis of measures such as overall pressure losses, point velocities and velocity variances and determining when a plateau has been achieved. All turbulent statistics data were calculated over the remaining 0.15 s, a period required for mean values to converge, sampled at every time step.

### 2.3 Utilised area

Table 3 shows UA data for several types of fully developed flow which have analytical solutions, all calculated for circular ducts.

## Figures and Tables

**Table 1:** Refinement statistics data for the finest simulation at a steady flow of 30 l.min^−1^.

	Reference Simulation (9.2 million elements, ∆*t*=0.01 ms)
Mean $y^{+}$	0.096
Maximum $y^{+}$	0.43
Mean $3\sqrt{V}/\eta$	2.36
Mean $∆t/\tau_{\eta }$	0.0336
Mean $U∆t/\tau_{\eta }$	0.83

**Table 2:** Pressure loss (∆P) data for reference and LES.

Simulation	Mean ∆P (Pa)	Amplitude of ∆P oscillations (Pa)	Approximate frequency of ∆P oscillations (Hz)
Reference	53.8	6.2	312
LES	53.8	6.3	312

**Table 3:** Comparison of utilised area ratios for several analytical flow profiles.

	Poiseuille	Turbulent Re=4000^a^	Turbulent Re=23000	De=50^b^	De=100	De=150	De=200
$\frac{{\mathrm{UA}}_{\max}}{A}$	0.5	0.970	0.980	0.498	0.462	0.397	0.292
$\frac{{\mathrm{UA}}_{\mathrm{mean}}}{A}$	0.5	0.568	0.571	0.499	0.489	0.451	0.419

a Schlichting [5]

b Doorly and Sherwin [3]
